# Evaluation of an advance care planning training program for practice professionals in Japan incorporating shared decision making skills training: a prospective study of a curricular intervention

**DOI:** 10.1186/s12904-022-01019-x

**Published:** 2022-07-26

**Authors:** Yuko Goto, Hisayuki Miura, Yasuhiro Yamaguchi, Joji Onishi

**Affiliations:** 1 Department of Home Care and Regional Liaison Promotion, National Center for Geriatrics and Gerontology, Nagoya, Japan; 2 Department of Respiratory Medicine, Jichi Medical University Saitama Medical Center, Saitama, Japan; 3 Department of Community Health Care and Geriatrics, Nagoya University Graduate School of Medicine, Nagoya, Japan

**Keywords:** Advance care planning, Shared decision making, Skills training, Education program

## Abstract

**Background:**

We developed a novel training program for health care professionals that incorporated shared decision making (SDM) skills training into an advance care planning (ACP) training course, the first in Japan. This study aimed to assess the training program’s impact on health care professionals’ knowledge, skill, attitudes, and confidence to initiate ACP.

**Methods:**

Using the novel Japanese educational program, we evaluated the effect of 8-month programs conducted eight community training sites of professionals who can practice ACP in a local area in Aichi Prefecture (the Aichi ACP Project). SDM skills training was provided during the workshops conducted in the ACP training course, and the participants’ satisfaction and understanding of the training were assessed. After the completion of two workshops, information on SDM skill results from the training and submitted assignments were collected anonymously from the training sites.

**Result:**

A total of 404 participants completed all education programs. After the first workshop, at least 95% of trainees stated that they were satisfied with the training and that it was useful for ACP practice. The evaluation of the results between the first and second workshops indicated improvement in SDM skills on some items of the SDM measures. In the second workshop, at least 90% of participants submitted implementation reports, and after the second workshop, a survey of confidence in ACP practice was administered, with responses indicating improvement. There were high levels of interest in education related to the ACP practices of oneself and others.

**Conclusions:**

This educational program can be an effective for developing professionals who can practice ACP with SDM skills.

**Supplementary Information:**

The online version contains supplementary material available at 10.1186/s12904-022-01019-x.

## Background

In recent years, global changes, such as increased numbers of treatment options, more uncertainty regarding treatment, and a greater diversity of patient values, have created a need for more support in medical care and care decision making. Furthermore, there has been an increased interest in advance care planning (ACP), a dialog that shares patient values and supports patient-centered medical treatment. ACP allows patients to live their lives as they choose, even when they require medical treatment and care [1]. ACP is important for both patients and specialists [2, 3] because it allows for building a rapport can be built with specialists to initiate discussions before the patient’s condition deteriorates, thus allowing an earlier selection of a wide range of treatment and care options based on the patient’s values and life goals. A survey of family members of patients admitted to the emergency care unit reported a reduced level of conflict in patients whose families had participated in advance discussions regarding future medical treatment and care with the patients compared to families who had not had these discussions [4]. In the United Kingdom, an end-of-life care strategy calls for discussing future medical treatment and care between health care professionals and patients approaching the end of life [5]. It points to the importance of discussions between patients, physicians, and health care regarding future medical treatment and care in the contexts of patient values and preferences [6]. An international ACP research organization proposed some guidance on how ACP should be implemented to benefit patients and a definition for ACP [7]. With the accelerated global implementation of ACP, the international definition of ACP has been considered, a concrete ACP practice model created, and outcomes benefiting patients clarified [7].

In 2017, ACP was internationally defined as a “process that supports adults at any age or stage of health in understanding and sharing their values, life goals, and preferences regarding future medical care” [8]. In Japan, as a national policy, the government has begun to establish a consultation system for medical treatment and care at the final stage of life to allow individuals to live as humanely as possible [9]. Although the dialog is important in ACP practice and should begin when the patient can still participate in such discussions, it has become clear that many physicians and nurses in Japan do not want to initiate discussions before the patient’s condition worsens [10]. Moreover, although the conversation between patients and specialists in the field of palliative care is [11], the practice of such is reportedly difficult, and few educational programs on ACP practice have been confirmed effective [12].

Unlike in Europe and the United States, there is no basic law or societal rules in Japan that protect the ethical rights of patients (i.e., patient-centered care) [13]. Thus, there is a need to introduce educational programs for ACP professionals that incorporate dialog skills in shared decision making (SDM) [14, 15], which is the pinnacle of patient-centered care, to develop medical treatment and care that focuses on the focus on patient values. SDM is a method in which (1) at least two parties, a patient and specialist, participate; (2) both parties share information; (3) both parties are aware of the options and the corresponding details; (4) both parties agree on the decision while sharing decision criteria; and the patient and specialist make decisions together, including on the elements [16]. Moreover, because the practice of SDM is used in interactions between patients, physicians, and health care professionals, SDM requires dialog skills training. The beneficial effects of SDM on patients have been demonstrated in terms of medical satisfaction, trust in specialists, increased understanding of treatment and care, decreased decision making conflict, and improved adherence [17, 18].

In Japan, where older adults make up 30% of the total population [19], the number of critically ill patients is increasing alongside the spread of the COVID-19 pandemic [20]. The Japan Geriatrics Society strongly urges the implementation of ACP practice at an early stage [21], and there is an urgent need to develop human resources/professionals who can practice ACP even earlier, focusing on patient values. To this end, an educational program for professionals who can practice ACP, including SDM skills training, must be developed and launched in Japan. In response to this situation, in 2020, a training program (Aichi Prefectural 2020 Practical ACP Professions Development Education Program: the Aichi ACP Project) incorporating the concepts of SDM and ACP was conducted for the first time in Japan. Therefore, the study aims to assess the training program’s impact on health care professionals’ knowledge, skills, attitudes, and confidence to initiate ACP.

## Methods

### Study design

This prospective study of a curricular intervention collected and used data from those who completed the 2020 Practical ACP Professions Development Education Program (the Aichi ACP Project) led by the Aichi Prefectural Government.

### Evaluation framework

This study validated the effectiveness of an educational program for developing professionals who will practice ACP incorporating SDM skills training using the New World Kirkpatrick Model [22–24]. The New World Kirkpatrick Model assesses the effectiveness of training programs at the following four levels: level 1, the trainee’s response to the training experience (including training experience); level 2, the learner’s learning outcomes and increases in knowledge, skill, and attitude toward the attendance experience (i.e., how much attendees learned the content after training, typically measured using a pretest and posttest); level 3, the students’ change in behavior and improvement (whether the learning transfers into practice in the workplace); and level 4, results (the ultimate impact of training).

Since the evaluation took place immediately after the training it was based on the first to the third level of Kirkpatrick’s model: reaction (level 1), learning (level 2), and behavior (level 3).

### Development of a new Japanese educational program for professionals who can practice ACP

The Aichi ACP Project is a training program “completed” via participation in two venue workshops and submitting before each workshop. The training was conducted at eight sites adopted by the Aichi Prefecture for the Aichi ACP Project. Each training site recruited participants and enrolled individuals from among medical, nursing, or welfare specialists in the field to support patient decision making. Enrolled participants completed preliminary learning via e-learning to allow for knowledge acquisition before the first training session. In the first workshop, SDM skills training was conducted using a dialog on ACP through lectures and role-playing.

The first workshop conducted role-playing for practical training of SDM with a small number of people. In addition, various professionals teamed up to discuss how to support difficult patient decisions in groups. There were two main instructors at the workshop, and four sub instructors provided participant support at each training site.

In the role-playing, we set up scenes in which some kind of health trouble occurred, with fictitious patients consisting of an older person with mild dementia living alone and an older person with dementia living with an unemployed and withdrawn family. Sets of three participants formed a team at random, and were divided into the roles of provider (decision supporter), patient, and observer. They then role-played SDM using the scenes. The individual who played the role of provider (decision supporter) drew on their own profession and expertise during the role-play to perform SDM. Immediately after role-playing, the three members evaluated the role-playing using the SDM measures [25, 26]. The participant playing the patient role evaluated the SDM received from the decision supporter role from the patient’s point of view. Three members then provided feedback on decision support and discussed ideas for improvement.

We set the target of group discussion as bedridden older patients with dementia who were receiving home care and family caregivers who could not tolerate the stress of long-term care. We set up a scene in which a patient had pneumonia and discussed the ACP practice with the collaboration of multiple professionals. The duration of the first workshop was 6 h.

In the questionnaire after the first workshop, we collected the respondents’ expertise, years of clinical experience, satisfaction with the Aichi ACP Project to date, and responses regarding the project’s difficulty as compared with their own prior predictions. To practice what was learned and learn from this experience before the second workshop, participants made efforts to practice ACP using SDM and perform regional and organizational development to enable practicing ACP in the organization or region to which they belonged. A report was then compiled, which was submitted at the second workshop.

Participants received SDM skills training during the second workshop using role-playing and group discussion. The second workshop was a 3-h session with two main instructors; no support sub instructor was included. The patient settings and scene settings for the role-playing in the second workshop were the same as for the first session of role-playing. As in the first workshop, three members formed a team at random and then role-played SDM using one situation by playing the roles of provider (decision supporter), patient, and observer. They evaluated their SDM skills, using SDM measurements and discussing improvement methods. The individual who played the provider (decision supporter) performed SDM using their own profession and expertise. Immediately after role-playing, the three members independently evaluated the role-playing using the SDM measure, provided feedback on decision support, and discussed improvements, as in the first workshop.

**Fig. 1:**
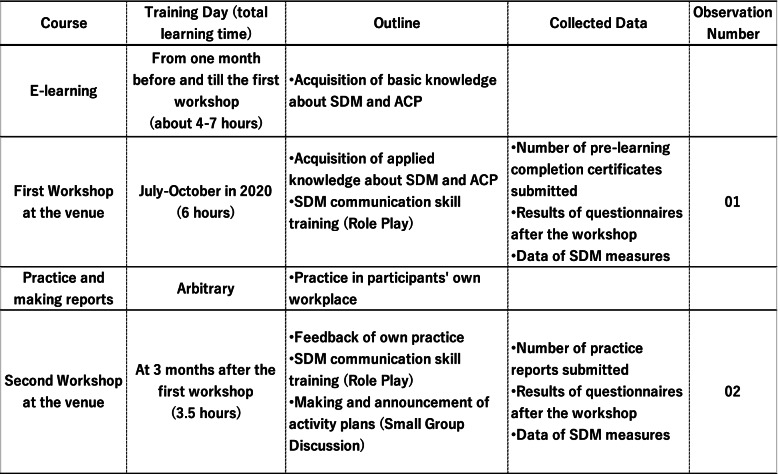
Aichi ACP Project educational program and data collection points

During the second workshop, participants presented practical reports on ACP and organizational development activities using SDM, which they had submitted as their assignment. They then drew up a draft ACP activity plan for the group. During the group discussion, the workshop participants then shared their ACP activity plans and devised an ACP practice plan on which the participants would work as a team in the future (Fig. 1).

In the questionnaire administered after the second workshop, we collected information on the respondents’ expertise, years of clinical experience, responses regarding the difficulty of the Aichi ACP Project compared with their advanced expectations, frequency of seeing the ACP practiced by other specialists, the possibility of using the Aichi ACP Project, and recognition of the necessity of continued learning regarding ACP in the future.

### Data collection

We collected data for usage anonymously from eight training sites of the Aichi ACP Project, which a government agency held in Aichi Prefecture, Japan, from July 2020 to February 2021. The eight training sites (seven hospitals and one organization promoting medical and long-term care coordination) completed all Aichi ACP Project programs. An individual in charge of information processing but not involved in this study entered the collected and anonymized data. The data included results of the questionnaire anonymously administered to participants after two sessions of venue workshops, SDM measurement data at workshops, and information on activity reports (Fig. 1).

Immediately after the first workshop, the participants provided the answers to the following items on the anonymous questionnaire: satisfaction with training, the frequency of seeing about patients’ ACP in the information provided by the liaison organizations, and frequency of seeing other people’s ACP practice. Answers were obtained using six-point Likert-type scales.

Immediately after the second workshop, the participants provided answers to the following items on an anonymous questionnaire at the venue: degree of confidence in ACP practice, frequency of seeing about patients’ ACP in the information provided by the liaison organizations, frequency of seeing other people’s ACP practice, the possibility of using the training content in their ACP practice, and the need for continuous ACP learning in the future. Answers were obtained using six-point Likert-type scales, and each result was replaced with a dummy variable ranging from 0 to 5.

### SDM measurement

**Fig. 2:**
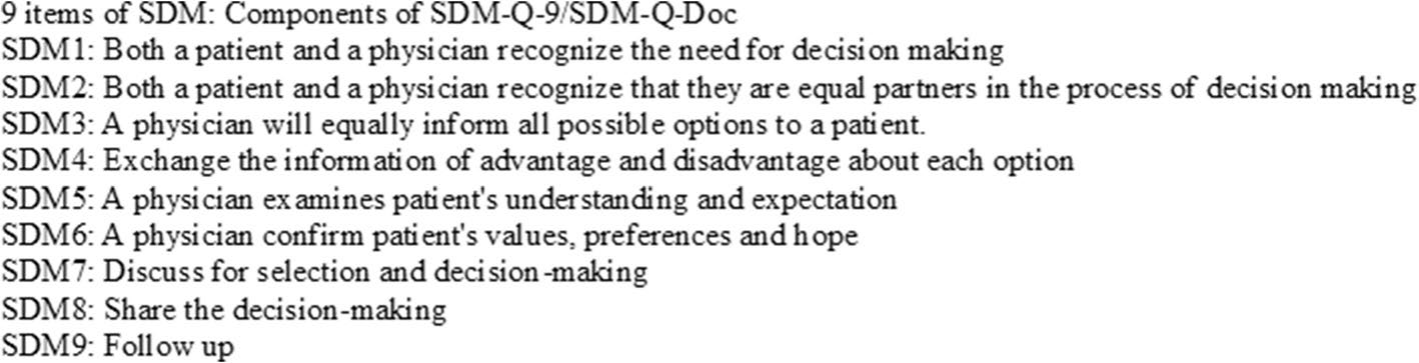
Nine items of SDM: components of the SDM-Q-9/SDM-Q-Doc

**Fig. 3:**
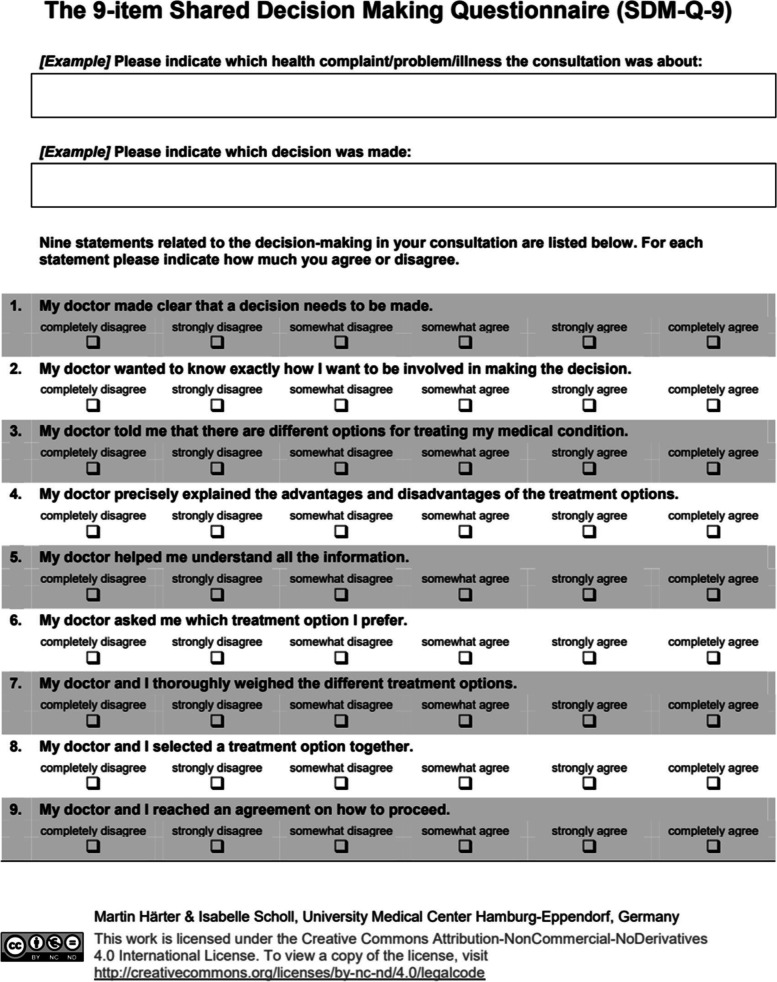
SDM-Q-9, English version

**Fig. 4:**
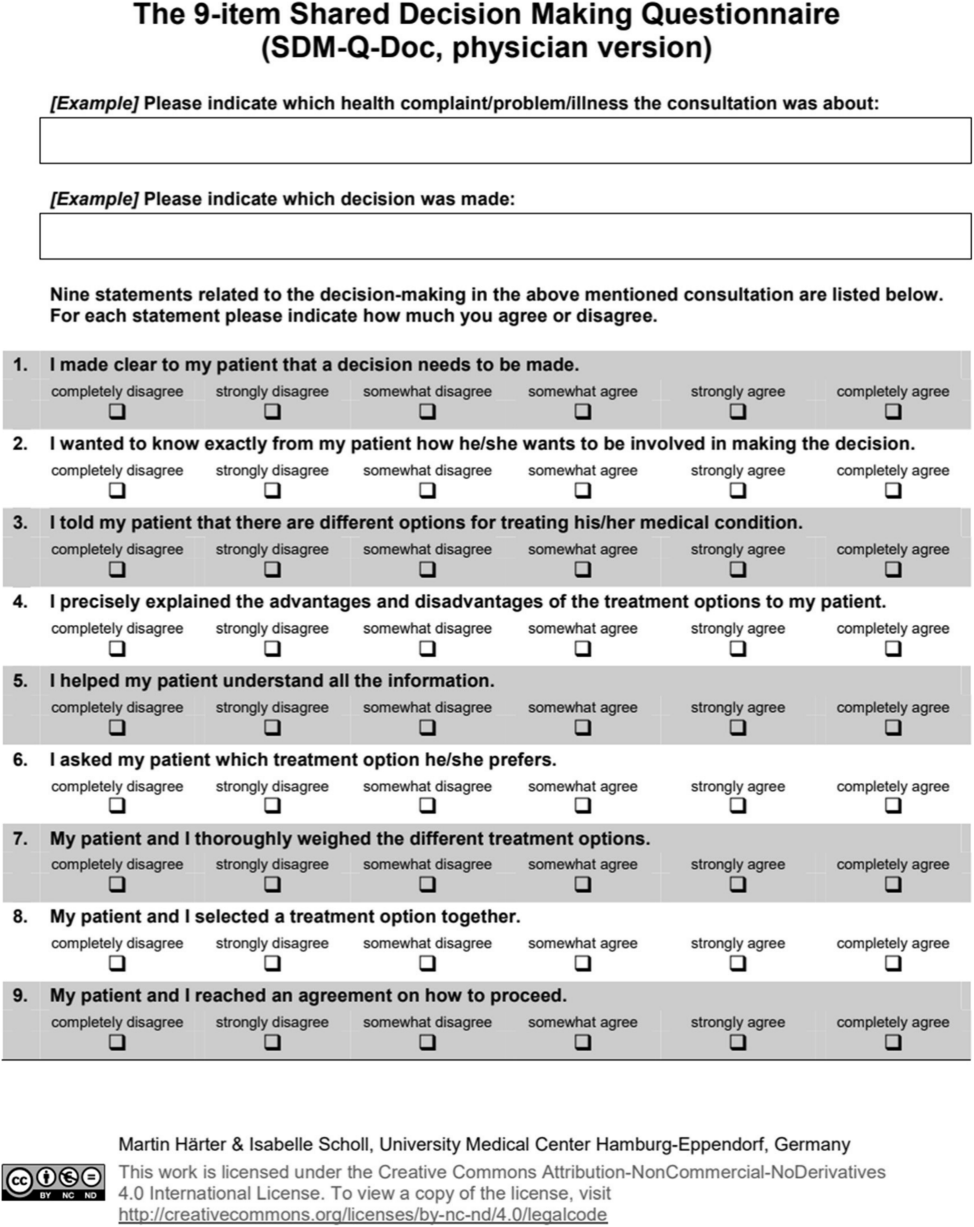
SDM-Q-Doc, English version

In order to evaluate SDM skills in this study, the SDM measure used in this study, namely, the SDM-Q-9 (patient) [25]/SDM-Q-Doc (physician) [26], was developed by the Department of Medical Psychology, University Medical Center Hamburg Eppendorf, Germany. It is the world’s first bidirectional SDM scale. The scale includes nine items that help visualize the degree of SDM (Figs. 2, 3, and 4). As of 2021, the measure had been translated into 29 languages [27]. Its reliability and validity are confirmed in various cultures and languages. The nine items of the SDM scale comprise a one-factor structure measuring the concept of SDM, and the Japanese version of the SDM-Q-9 (patient) [28]/Japanese version of the SDM-Q-Doc (physician) [29] has already been confirmed to be reliable and valid in clinical practice in Japan. Furthermore, the Japanese version of the SDM-Q-9 (patient)/Japanese version of the SDM-Q-Doc (physician) was adapted to create an SDM-C–Patient/SDM-C–Provider for use by health care professionals other than physicians. In addition, the configural and measurement invariance of the Japanese version of the SDM-Q-9(patient)/Japanese version of the SDM-Q-Doc (physician) and the SDM-C–Patient/SDM-C–Provider for use by care professionals has been confirmed [30]. All nine items are rated on a six-point Likert-type scale, where 0 corresponds to *completely disagree* and 5 corresponds to *completely agree*; a perfect score is 45 points.

Workshop participants were asked to fill out an anonymous SDM measurement form at the workshop venue.

### Statistical and qualitative analyses

#### SDM skills data and analysis

We compiled data from the two questionnaires from all training sites of the two workshops and analyzed the descriptive statistics. The evaluation of SDM skill was analyzed descriptively by substituting the scores for the SDM-Q-9/SDM-Q-Doc and the SDM-C–Patient (care patient)/SDM-C–Provider (care provider) from a perfect score of 45 to a perfect score of 100. We compared SDM skills between O1 and O2 (observation points in Fig. 1), with Wilcoxon rank-sum tests because the team members and roles in the role-play differed between O1 and O2, and we set *p* ≤ 0.05 as the level of significance. The sample size was calculated as an unpaired sample using a Wilcoxon rank-sum test [31]. With α = 0.05 and power = 0.8, the test being two-sided, and the effect size is set at a moderate *d* = 0.5 because there were no prior studies, we calculated the number in each group as 67.

#### Analysis of questionnaire immediately after training

We confirmed the difference between the difficulty levels after the first and second workshops using a chi-squared test, and the level of statistical significance was set as *p* ≤ 0.05. We analyzed the data from the questionnaire results after the second workshop using a covariance structure analysis. The perceptions from the training and environmental factors influencing the trainees were clarified. Using chi-squared values, we evaluated the model’s goodness-of-fit with the root mean square error of approximation (RMSEA), goodness-of-fit index (GFI), the adjusted goodness-of-fit index (AGFI), and the comparative fit index (CFI).

#### Analysis of SDM reports and organizational/regional development activity reports enabling the practice of ACP

In the SDM reports, we extracted key concepts related to promoting and inhibitory factors for clinical practice of SDM; in turn, key concepts with a number and frequency of occurrence > 10% of the total were also extracted.

The key concepts of the activity were extracted from the organizational/regional development activity reports enabling the practice of ACP, and, in turn, the key concepts with an appearance frequency > 10% were extracted. In addition, we also extracted the promoting and inhibitory factors and key concepts exceeding 10% of the total.

#### For statistical analysis, we used IBM SPSS Statistics 28 and IBM SPSS Amos Graphics 28

We performed a computerized lexical analysis of free text from the SDM reports and organizational/regional development activity reports enabling the practice of ACP, using the Japanese version of SPSS Text Analytics for Surveys 4.0.1. The participants’ reports were imported into the software, which extracts key terms used to categorize the responses. To create categories for this project, we combined both linguistic and frequency algorithms in the software. Once the data were categorized, the individual reports responses and the associated categorized data were exported for subsequent data analysis by several researchers. Through this process, the core sentences containing frequently occurring words were extracted, and the participants’ description contents were analyzed to create categories for this project.

## Results

### Participant characteristics

Although 445 individuals participated in the first workshop (O1), 404 (91%) completed all Aichi ACP Project programs, including the second workshop (O2). In terms of profession, nurses accounted for at least 40% of all participants, and medical social workers, care support specialists, and physicians accounted for about 10% each (Table 1). Most of the professionals in the program had more than > 25 years of clinical experience, followed by those with 10 years to < 15 years of clinical experience (Table 2).

**Table 1:** Profession of Participants (*n* = 445 in O1, *n* = 404 in O2)

	Number (%)	
Occupation	O1: First Workshop	O2: Second Workshop
Nurse	187 (42)	176 (44)
Medical social worker	70 (16)	57 (14)
Care manager	56 (13)	53 (14)
Physician	47 (11)	41 (10)
Pharmacist	29 (6)	29 (7)
Dentist	10 (2)	9 (2)
Therapist	8 (2)	5 (1)
Public health nurse	5 (1)	5 (1)
Others and no answer	33 (7)	29 (7)
Total	445 (100)	404 (100)

**Table 2:** Clinical experience of participants ( *n* = 445 in O1, *n* = 404 in O2)

	Number (%)	
Years of clinical experience (years)	O1: First Workshop	O2: Second Workshop
< 5 years	41 (9)	44 (11)
5 years to < 10 years	65 (14)	56 (14)
10 years to < 15 years	88 (20)	70 (17)
15 years and < 20 years	70 (16)	66 (17)
20 years to < 25 years	70 (16)	66 (17)
> 25 years	102 (23)	94 (23)
Others/ no answer	9 (2)	8 (1)
Total	445 (100)	404 (100)

### Training evaluation

#### Level 1 reaction

The first workshop had 445 participants, and 438 submitted the questionnaire afterward (response rate = 98%). From the questionnaire results after the first workshop, 22% were very satisfied, 52% were satisfied, and 23% were a little satisfied (Table 3).

**Table 3:** Satisfaction with the ACP education program (*n* = 438 in O1)

	Number (%)
Very satisfied	91(21)
Satisfied	228(52)
A little satisfied	100(23)
A little not satisfied	13(3)
Not satisfied	1(0)
Not satisfied at all	0(0)
No answer	5(1)
Total	438(100)

#### Level 2 learning

We compared the results of SDM role-play via dialog between the first workshop (O1) and the second workshop (O2) using a Wilcoxon rank-sum test. The pairs in which the physician/dentist acted as the provider (decision supporter) role-played treatment decisions using the Japanese version of the SDM-Q-9 (patient)/Japanese version of the SDM-Q-Doc (physician). The pairs in which the care provider played the role of the provider (decision supporter) role-played care decisions using the SDM-C–Patient/SDM-C–Provider. Only data without missing values and connected data from the role-play of the patient and provider were included in the analyses. In the first workshop (O1), data from 145 pairs (provider-patient = 145) were included in the analysis. For the second workshop (O2), 131 pairs were included (Provider = Patient = 131).

### SDM for those playing the role of patients

We compared the SDM data for patients between observation points O1 and O2 using a Wilcoxon rank-sum test (Tables 4 and 5).

**Table 4:** Descriptive statistics of the patient SDM scores at observation points O1 and O2

		Patient								
		SDM1	SDM2	SDM3	SDM4	SDM5	SDM6	SDM7	SDM8	SDM9
O1	Median	8.89	8.89	8.89	6.67	8.89	8.89	6.67	8.89	8.89
	Minimum	2.22	2	0	0	0	0	0	0	0
	Max	11.11	11.11	11.11	11.11	11.11	11.11	11.11	11.11	11.11
	Mean	8.6	8.26	8.38	6.8	8.25	8.6	6.65	8.05	8.15
	Standard deviation	2.13	2.28	2.5	2.76	2.27	2.52	2.55	2.68	2.85
O2	Median	8.89	8.89	8.89	6.67	8.89	8.89	6.67	8.89	8.89
	Minimum	2.22	0	2.22	0	0	0	2.22	2.22	0
	Max	11.11	11.11	11.11	11.11	11.11	11.11	11.11	11.11	11.11
	Mean	8.58	8.23	8.69	7.11	8.36	9.19	7.32	8.52	8.75
	Standard deviation	1.98	2.15	2.24	2.68	2.11	2	2.39	2.28	2.31

**Table 5:** Comparison of patient SDM scores at observation points O1 and O2 (Wilcoxon rank-sum test)

	Patient								
	SDM1	SDM2	SDM3	SDM4	SDM5	SDM6	SDM7	SDM8	SDM9
W	18,115.0	20,042.0	19,203.0	19,731.0	20,052.5	18,980.5	18,716.0 ^*^	19,331.5	18,939.0
Z	− 0.046	− 0.064	− 1.395	− 0.545	− 0.048	− 1.758	− 2.138	− 1.180	− 1.806
*p*	0.964	0.949	0.163	0.586	0.962	0.079	0.032	0.238	0.071

^*^ *p* ≤ 0.05

The nine items of the SDM measure were rated on a six-point Likert-type scale. The role-playing patient role evaluated the SDM received from the decision supporter role on a nine-point scale using the Japanese version of the SDM-Q-9 (patient) or SDM-C–Patient (care patient). Participants evaluated each item on a six-point scale, where 0 corresponds to *completely disagree* and 5 corresponds to *completely agree*; a perfect score was 45 points. For each item, we multiplied by 20/9 so that the total of nine items would be 100 points. In addition, we summarized the descriptive statistics for each item (Table 4). The difference between O1 and O2 was analyzed using the Wilcoxon rank-sum test, and as a result, the SDM measure SDM7 (discuss for selection and decision making) increased significantly (Table 5).

### SDM for those playing the role of providers

**Fig. 5:**
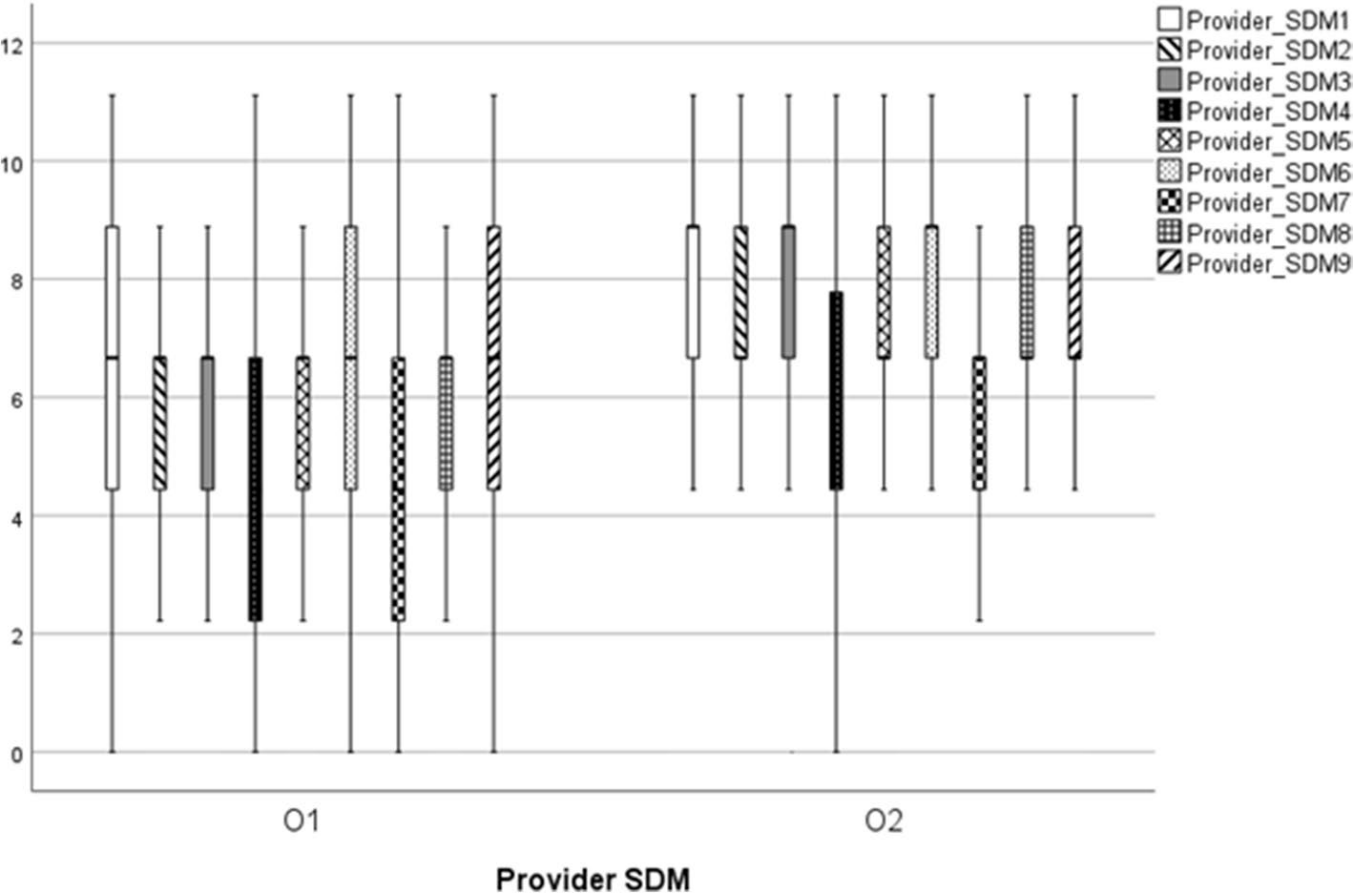
SDM scores of the providers at observation points O1 and O2 (box-and-whisker plots)

SDM data for providers were compared between observation points O1 and O2 using a Wilcoxon rank-sum (Fig. 5; Tables 6 and 7).

**Table 6:** Descriptive statistics of provider SDM scores at observation points O1 and O2

		Provider								
		SDM1	SDM2	SDM3	SDM4	SDM5	SDM6	SDM7	SDM8	SDM9
O1	Median	6.67	6.67	6.67	4.44	6.67	6.67	4.44	6.67	6.67
	Minimum	0	0	0	0	0	0	0	0	0
	Max	11.11	11.11	11.11	11.11	11.11	11.11	11.11	11.11	11.11
	Mean	6.77	6.01	6.36	5.15	6.16	6.51	4.84	5.93	6.21
	Standard deviation	2.53	2.29	2.37	2.55	2.07	2.7	2.38	2.58	2.66
O2	Median	8.89	6.67	6.67	6.67	6.67	8.89	6.67	6.67	6.67
	Minimum	0.00	0.00	0.00	0.00	2.22	2.22	0.00	0.00	0.00
	Max	11.11	11.11	11.11	11.11	11.11	11.11	11.11	11.11	11.11
	Mean	7.79	7.05	7.53	6.13	6.93	8.89	5.80	7.15	7.50
	Standard deviation	2.23	2.13	2.04	2.23	1.80	1.97	1.91	2.05	2.20

The decision making supporter role evaluated their SDM on nine items during the role-playing, using the SDM-Q-Doc (physician) or SDM-C–Provider (care provider). The participants evaluated the items on a six-point scale, where 0 corresponds to *completely disagree* and 5 corresponds to *agree completely*; a perfect score was 45 points. For each item, we multiplied by 20/9 so that nine items would be 100 points. The descriptive statistics for each item were also summarized (Fig. 5; Table 6).

We analyzed the difference between O1 and O2 using the Wilcoxon rank-sum test. We found that the scores of all nine SDM items increased significantly. The median values for SDMs 1, 4, 6, and 7 increased (Table 7).

**Table 7:** Comparison of provider SDM scores at observation points O1 and O2 (Wilcoxon rank-sum test)

	Provider								
	SDM1	SDM2	SDM3	SDM4	SDM5	SDM6	SDM7	SDM8	SDM9
W	17,760.0 ^*^	17,688.0 ^*^	17,000.0 ^*^	17,654.0 ^*^	18,319.0 ^*^	17,667.0 ^*^	17,945.5 ^*^	17,713.0 ^*^	17,522.5 ^*^
Z	− 3.66	− 3.82	− 4.92	− 3.81	− 2.92	− 3.84	− 3.39	− 3.77	− 4.06
*P*	< 0.001	< 0.001	< 0.001	< 0.001	0.002	< 0.001	0.001	< 0.001	< 0.001

^*^ *p* ≤ 0.05

#### Level 3 behavior

Of the 404 participants, 402 submitted practical SDM reports and organizational/regional development activity reports enabling ACP practice at the second workshop. From the practical SDM reports, we extracted 819 key concepts as promoting factors for decision making. Half of these were “other specialists who understood this activity and helped their decision making support.” The next most common was “patients and their families were able to discuss decision making,” accounting for 30% of the overall total. In addition, “a trusting relationship with the patient/family has been established,” and “a room and time to support decision making can be secured” were mentioned. Moreover, we identified 645 key concepts as obstacles to implementing decision support. “No time or room” was the most common, accounting for 20% of the overall total. Next, was “patients or their families were in situations where dialogs for decision making were challenging due to the comorbidity of dementia or other reasons,” accounting for about 20% of the overall total. Furthermore, a “lack of knowledge and communication skills of specialists to support decision making” accounted for about 10% of the total (Table 8).

**Table 8:** Promoting and inhibitory factors for clinical practice of SDM extracted from the SDM reports

Promoting factors (*n* = 819)	Inhibitory factors (*n* = 645)
Other specialists understood this activity and helped their decision making support (50%)	No time or room (20%)
Patients and their families were able to discuss decision making (30%)	Patients or their families were in situations in which dialogs for decision making were challenging because of the comorbidity of dementia or another reason (20%)
	Lack of knowledge and communication skills of specialists to support decision making (10%)

We extracted 311 activity key concepts from the organizational/regional development activity reports that enabled ACP practice. The most common category was “ACP training was provided for multiprofessions, who did not join the Aichi ACP Project, in their workplaces and region, accounting for 25% of the total, followed by “ACP educational activities for concerned parties, residents, and patients.” Moreover, skills learned were often used in actual clinical practice, and “reflection, etc. at conferences where concerned parties gathered” was often performed. We extracted 561 key concept samong factors promoting organizational/regional development allowing ACP to be practiced. The most common key concept was “presence of a supervisor or colleague who understands the need to practice ACP in my workplace or region,” accounting for 40% of all key concepts. Next was the “presence of a cooperative system that enables us to work together in the organization or region,” accounting for 20% of all key concepts. Moreover, participants mentioned “in an environment where I am involved with patients, etc., who need ACP support,” “presence of some individuals interested in ACP,” and “there is an opportunity to learn about ACP.” As obstacles in organizational/regional development allowing ACP to be practiced, 541 key concepts were extracted. The most common key concept was “inability to gain the understanding and cooperation of individuals involved in my workplace or region,” which accounted for 40% of all key concepts. This was followed by “not enough time/too busy,” which accounted for about 30% of the total (Table 9). In addition, a “lack of knowledge, skills, or ability to communicate” of individuals completing the Aichi ACP Project themselves was mentioned.

**Table 9:** Promoting and inhibitory factors extracted from organizational/regional development activity reports enabling the practice of ACP

Promoting factors (*n* = 561)	Inhibitory factors (*n* = 541)
Presence of a supervisor or colleague who understands the need to practice ACP in my workplace or region (40%)	Inability to gain the understanding and cooperation of individuals involved in my workplace or region (40%)
Presence of a cooperative system that enables us to work together in the organization or region (20%)	Not enough time/Too busy (30%)

The results of the questionnaire administered after the second workshop revealed the following responses to the questions regarding confidence toward future implementation of ACP: “very confident” (1%), “gained confidence” (11%), and “if anything, gained confidence” (73%). At present, 60% of those who completed the program answered that they often see information about the patient’s sense of values when they receive information from other institutions supporting hospitalization and discharge, whereas 46% answered that they see other individuals practicing ACP. Among those who completed the program, 96% answered that they could use the skills and knowledge acquired from the programs in the Aichi ACP Project. Among all respondents, 98% stated they felt the need to continue learning about ACP.

### Path analysis using structural equation modeling of participants’ perceptions and awareness

**Fig. 6:**
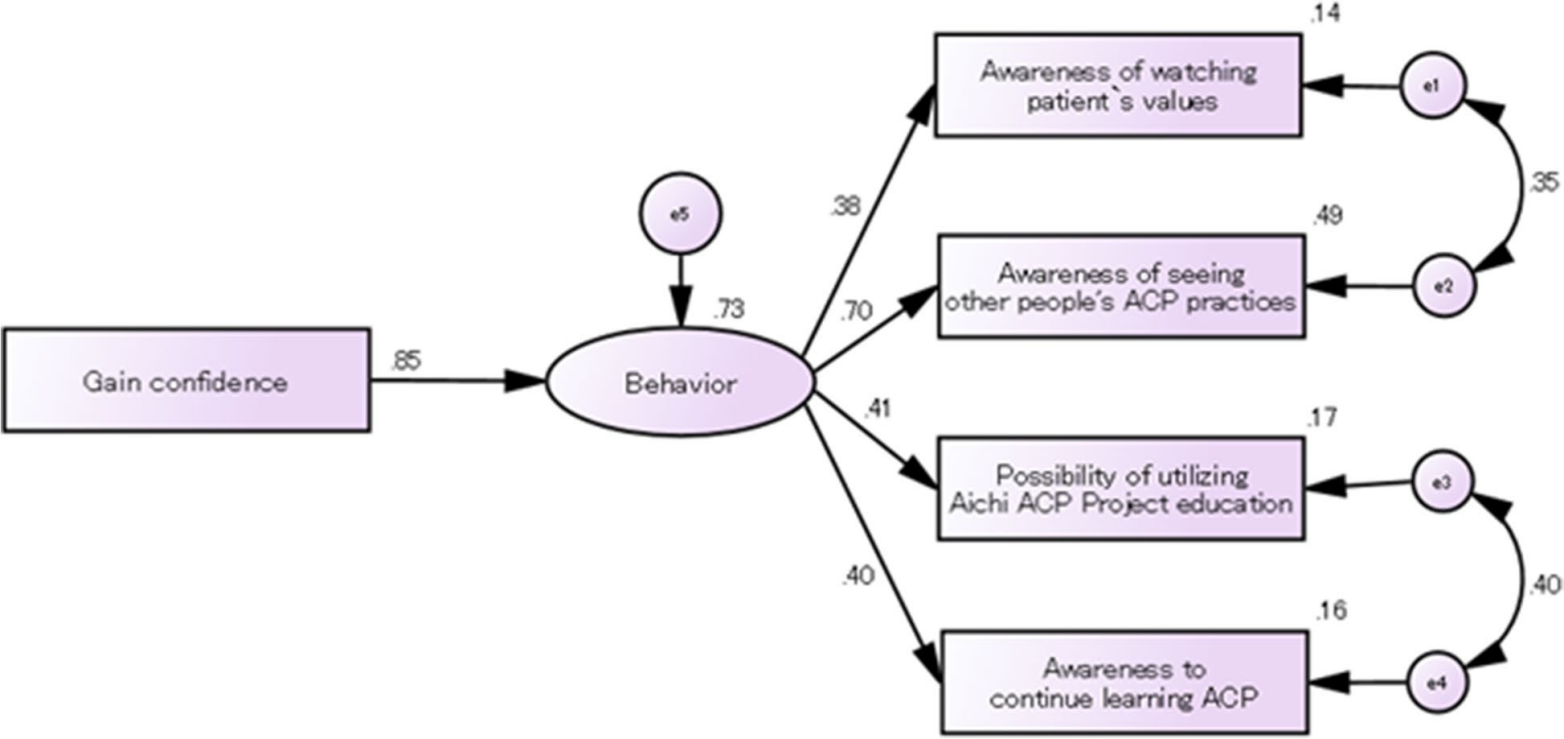
Path analysis using structural equation modeling of participants’ perceptions and awareness

We analyzed the relationship between the practice and perception of individuals who completed using structural equation modeling. Based on these results, we estimated that there were some changes in confidence. This affected the perspective of seeing information regarding the patient’s sense of values, the perspective of seeing others practice ACP, the expectation regarding the use of educational content of the Aichi ACP Project, and the self-awareness of the need for continuous learning (Fig. 6). The goodness-of-fit of the model was as follows: χ^2^ = 4.498 (*p* = 0.212), RMSEA = 0.035, GFI = 0.996, AGFI = 0.978, CFI = 0.997.

## Discussion

### Training evaluation for the Aichi ACP Project

Until recently, ACP training that incorporates SDM skills has not been conducted in Japan, despite its importance. The importance of SDM in ACP practice has been recognized worldwide, but there are only limited reports of training programs that integrate SDM and ACP [32, 33]. Goossens et al. [32, 33] reported the effect of a training program for nursing home staff on SDM skills in dementia care. In this study, we evaluated the effectiveness of the educational program, the Aichi ACP Project, for the development of professionals to practice ACP that incorporated SDM skills training for the first time in Japan. In this program, we applied the empirical learning theory [34], which theorizes the establishment of knowledge and skills through repeated learning and experiences. The educational program for the Aichi ACP Project consisted of training and understanding of ACP and basic dialog; training in SDM support skills, which is a patient-centered care method; and an ACP educational program that encompasses the knowledge and skills to practice ACP in a team.

The presence of professionals who can facilitate ACP for high-quality ACP practice is important [35], and programs in which professionals receive development resources to facilitate ACP and its educational evaluation are indispensable. The novelty of this study lies in the systematic evaluation of educational effects from level 1 (reaction) to level 3 (behavior) using the New World Kirkpatrick Model. In previous ACP education studies, the major domains of evaluation included dialog training [36], involvement in patient advocacy [37], and behavior change of the trainee [38]. Very few studies have provided a systematic evaluation of the education effect.

Below, we discuss each level of the New World Kirkpatrick Model.

### Level 1: reaction

Because the participants indicated high satisfaction on the questionnaire after the first training workshop (O1), the training content appeared to be adapted to their training needs.

### Level 2: learning

In evaluating the acquisition of SDM skills, the results indicated that the provider, who is the decision supporter, showed relatively increased skills. However, the results of the role of the patient showed increased skills in only 1 of the 9 SDM items and did not generally show sufficient improvement in skills. There are no skills training programs for decision making support in basic education courses for medical care, nursing care, or welfare professionals in Japan. This may have been why many participants acquired knowledge of SDM, experienced skills, and learned practice for the first time in the Aichi ACP Project. Because many participants completed the Aichi ACP Project, considering that O2 is more difficult than O1, we thought that they understood the difficulty of learning and understanding. Because the participants recognized the need to learn about ACP at the end of O2 continuously, we anticipate that skills training will be provided continuously. On the other hand, the results showing that SDM7 (i.e., discuss for selection and decision making) significantly improved in the role of the patient is considered to be an important result for Japanese specialists. In the proceeding SDM, a three-talk mode [39] is being proposed, consisting of a “team talk,” “option talk,” and “decision talk.” In particular, the “option talk,” which includes “risk communication,” is an important discussion for patients and specialists to mutually exchange information about the merits and demerits of options and select options based on their understanding. Previous research showed that patients cannot always consult with health care professionals in Japan [40]. Patients can now discuss information regarding their options. ACP education has a significant educational effect that supports life based on the patient’s values. Although the number of previous studies introducing SDM into ACP educational programs is still limited nationwide, its importance has been confirmed [41]. In addition, although there has been little progress in developing a system for promoting SDM at the organizational and regional levels, its necessity has been demonstrated [42]. When practicing ACP, it is important to approach patients and their families, stakeholders, and teams to support decision making using SDM [43].

### Level 3: behavior

Our results showed that both the practice of ACP and the willingness to learn more about ACP have increased. For individuals to play an active role in ACP practice in community-integrated care, it is important that they continuously practice ACP. Thus, the education program in the Aichi ACP Project appears to be a valid and effective education program for regional development to practice ACP in comprehensive regional care.

In the assessment of the participants’ behavioral change, the most common promoting factor cited was the “ability to gain the understanding and cooperation of individuals involved in my workplace or region,” according to a practical ACP report through SDM and organizational/regional development activity reports. Thus, providing ACP education in small areas for multidisciplinary professionals who support patients can be practically effective for nurturing and promoting ACP in professions and activities. In addition, according to these reports, “no time or room” and “lack of knowledge and skills of the specialist” were identified as common inhibitors. Thus, organized support is needed to secure time and venues. Moreover, as described above, continuous skills training and educational opportunities have also been suggested for a “lack of knowledge and skills of the specialists themselves. Logistics support such as securing organizational training opportunities for organization managers and securing time and venues are also required.

### Comprehensive evaluation from levels 1 to 3

Individuals who completed the training were considered to have acquired the necessary decision making support skills for practicing ACP, the confidence to practice ACP in the future, and a perspective encompassing the need to continuously learn about ACP and be aware of information on patient values and of others practicing ACP. Such consciousness and awareness should promote changes in the behavior of practicing ACP professionals. Thus, the Aichi ACP Project is an effective educational program for developing professionals who can practice ACP.

### Study limitations

This study was carried out as an independent project led by the Aichi Prefectural Government. Thus, within the framework of the project, there was no opportunity for specialists from regions other than Aichi Prefecture to undergo training in the Aichi ACP Project. Therefore, there is a possibility of bias in the participation data may have been biased.

### Feasibility of the program

Another limitation of this study is that although various training sessions using venues were suspended or postponed because of the nationwide spread of COVID-19 infection, it was necessary to consider implementing a long-term educational program centered on venue training for medical and long-term care professions. The Aichi ACP Project was an effective educational program that encouraged participants to improve their skills and change their consciousness. Thus, there is a strong requirement for infection control and an online educational program so that people in various regions can obtain educational opportunities in the future.

## Conclusions

We confirmed the ACP education program by Aichi Prefecture improved the quality of the SDM skills of participants and enabled them to gain confidence in practicing ACP and become more conscious of their own and others’ practice of ACP. It increased their motivation to engage in continuous learning. This ACP education program is considered effective for developing ACP area leaders.

## Supplementary Information

**Additional file 1.** 

## Data Availability

The data sets used and/or analyzed during the current study are available from the corresponding author on reasonable request.

## Abbreviations

SDM: Shared decision making
ACP: Advance care planning
SDM-Q-9: The nine-item Shared Decision Making Questionnaire
SDM-Q-Doc: The Shared Decision Making Questionnaire—physician version
SDM-C–Patient: SDM-C–patient (care patient)
SDM-C–Provider: SDM-C–provider (care provider)
Aichi ACP Project: The Aichi Training Project for the Advance Care Planning Area Leader
