# Correction: Evaluation of an advance care planning training program for practice professionals in Japan incorporating shared decision making skills training: a prospective study of a curricular intervention

**DOI:** 10.1186/s12904-022-01036-w

**Published:** 2022-08-16

**Authors:** Yuko Goto, Hisayuki Miura, Yasuhiro Yamaguchi, Joji Onishi

**Affiliations:** 1 Department of Home Care and Regional Liaison Promotion, National Center for Geriatrics and Gerontology, Nagoya, Japan; 2 Department of Respiratory Medicine, Jichi Medical University Saitama Medical Center, Saitama, Japan; 3 Department of Community Health Care and Geriatrics, Nagoya University Graduate School of Medicine, Nagoya, Japan

**Correction: BMC Palliat Care 21, 135 (2022)**

**https://doi.org/10.1186/s12904-022-01019-x**

Following the publication of the original article [1], the author reported that the corrections concerning the correct sequence of the reference list were not carried out.

The original article has been corrected.
